# Inhibition of hippocampal or thalamic inputs to the nucleus accumbens reverses stress-induced alterations in dopamine system function

**DOI:** 10.1093/ijnp/pyaf034

**Published:** 2025-05-23

**Authors:** Hannah B Reiley, Alexandra M McCoy, Angela M Boley, Olivia J Yang, Natalie I Belle, Daniel J Lodge

**Affiliations:** Department of Pharmacology, Center for Biomedical Neuroscience, University of Texas Health Science Center, San Antonio, TX, United States; Department of Pharmacology, Vanderbilt University, Nashville, TN, United States; Vanderbilt Center for Addiction Research, Vanderbilt University, Nashville, TN, United States; Department of Pharmacology, Center for Biomedical Neuroscience, University of Texas Health Science Center, San Antonio, TX, United States; Department of Pharmacology, Center for Biomedical Neuroscience, University of Texas Health Science Center, San Antonio, TX, United States; Department of Pharmacology, Center for Biomedical Neuroscience, University of Texas Health Science Center, San Antonio, TX, United States; Department of Biology, College of Wooster, Wooster, OH, United States; Department of Pharmacology, Center for Biomedical Neuroscience, University of Texas Health Science Center, San Antonio, TX, United States; South Texas Veterans Health Care System, Audie L. Murphy Division, San Antonio, TX, United States

**Keywords:** psychosis, dopamine, hippocampus, thalamus

## Abstract

**Background:**

Symptoms of psychosis are often observed in patients with post-traumatic stress disorder (PTSD) and are driven by aberrant regulation of the mesolimbic dopamine system. We have previously shown that targeting upstream brain regions that regulate dopamine neuron activity, the ventral hippocampus (vHipp), and paraventricular nucleus of the thalamus (PVT) maybe a novel approach to restore dopamine system function. The vHipp and PVT work in concert to regulate ventral tegmental area (VTA) dopamine neuron activity through a multisynaptic circuit that begins with inputs to the nucleus accumbens (NAc). Therefore, we hypothesized that inhibition of projections from either the vHipp or PVT to the NAc would reverse stress-induced alterations in dopamine system function.

**Methods:**

In this study, we induced stress-related pathophysiology in rats using a 2-day inescapable foot shock procedure. We then examined if foot shock stress altered the firing patterns and coordinated neuronal activity within vHipp and PVT circuits. Finally, we examined if chemogenetic inhibition of NAc afferents could reverse stress-induced alterations in dopamine system function.

**Results:**

We observed a significant increase in coherence between the PVT and NAc up to 48 hours after foot shock stress. In addition, stress increased VTA dopamine neuron population activity, which was reversed following chemogenetic inhibition of either vHipp-NAc or PVT-NAc projections.

**Conclusions:**

Taken together, these results suggest that increased coherence between the PVT and NAc, following stress, may contribute to psychosis-like symptoms but targeting either the PVT or vHipp may be viable options for the treatment of comorbid psychosis related to PTSD.

**Significance Statement**

In addition to hallmark symptoms of post-traumatic stress disorder (PTSD), many patients experience symptoms of psychosis, including delusions and hallucinations. Antipsychotic medications can effectively lessen symptoms of psychosis, but do so by targeting dopamine receptors directly, and result in adverse side effects and poor patient compliance. Thus, targeting upstream brain regions that regulate dopamine neuron activity, such as the ventral hippocampus (vHipp) or paraventricular nucleus of the thalamus (PVT), may provide a therapeutic approach that has fewer adverse effects. We found that inescapable foot shock increased coordinated activity between the PVT and the nucleus accumbens and that inhibition of either the vHipp or PVT reversed stress-induced alterations in dopamine system function, suggesting that either region may be a novel site of intervention for comorbid psychosis related to PTSD.

## INTRODUCTION

The stress response is a physiological, adaptive process that helps individuals respond appropriately to their environment; however, prolonged or traumatic stressors can result in pathological alterations to neural circuits commonly associated with psychiatric disorders.^1^ One such system that is modulated by stressful or aversive stimuli is the mesolimbic dopamine system.^2-4^ Indeed, exposure to aversive stimuli, such as foot shock or tail shock, results in significant increases in extracellular dopamine within the nucleus accumbens (NAc).^5,6^ Both acute and chronic stressors can produce a lasting influence on mesolimbic dopamine neuron activity. Chronic stressors that are commonly used to study aspects of depression, such as prolonged exposure to cold or chronic mild stress, decrease ventral tegmental area (VTA) dopamine neuron population activity, defined as the number of spontaneously active dopamine neurons, via a pathway involving the basolateral amygdala.^7-9^ Conversely, acute stressors, such as foot shock or restraint stress, are used to model exposure to a singular traumatic event, such as those that precede symptoms of post-traumatic stress disorder (PTSD), and have been shown to increase VTA dopamine neuron population activity hours to days after exposure to the aversive stimulus.^4,10,11^ Increases in dopamine neuron population activity are also thought to underlie symptoms of psychosis, a notion that is supported by clinical data, in which elevated striatal dopamine transmission correlates with psychosis symptom severity.^12^ Interestingly, symptoms of psychosis in patients diagnosed with PTSD are common,^13-18^ suggesting that exposure to a traumatic event may have a profound influence on mesolimbic dopamine transmission, yet the underlying pathology that contributes to hallucinations and delusions following stress is not well understood.

Although increased dopamine transmission is thought to contribute to hallucinations and delusions, no clear histopathology has been identified within the mesolimbic dopamine neurons of patients with psychosis.^12,19^ Rather, the stress-induced alterations in neural activity appear to originate in brain regions that regulate dopamine neuron activity, such as the ventral hippocampus (vHipp) and the paraventricular nucleus of the thalamus (PVT).^11,20-22^ Both hippocampal and thalamic abnormalities are common observations in patients with PTSD as well as patients with psychosis.^23-27^ Indeed, decreased hippocampal volume is a hallmark of PTSD and alterations in hippocampal activity are thought to underlie cognitive and emotional processing deficits observed in patients with PTSD.^28,29^ While both increases and decreases in vHipp activity have been observed following stress^30^ the PVT displays robust activation following exposure to physiological and psychological stressors, including foot shock, predator scent exposure, and restraint stress.^30-36^ Given that the vHipp and PVT are stress-sensitive regions that have been shown to influence VTA dopamine neuron population activity, either may be a viable target for alleviating symptoms of psychosis in PTSD.^20,21,30,33,34,37^

Activation of either the vHipp or PVT can increase VTA dopamine neuron population activity through a multisynaptic pathway that begins with glutamatergic projections to the NAc.^22,38-41^ Previous work from our laboratory has demonstrated that glutamatergic projections from the vHipp and PVT converge onto individual medium spiny neurons within the NAc and the ability of the vHipp and PVT to modulate VTA population activity requires concurrent activity from both regions.^22^ Specifically, we have previously demonstrated that chemogenetic activation of either vHipp-NAc or PVT-NAc projections significantly increases VTA dopamine neuron population activity without affecting the firing rate or bursting pattern.^21,22^ Furthermore, pharmacological or chemogenetic inhibition of either the vHipp or PVT, in rodent models used to study aspects of psychosis, can reverse aberrant dopamine system function.^11,22,42,43^ Therefore, targeting either region may be a novel site of intervention for comorbid psychosis related to PTSD.

Recent work from our laboratory has also demonstrated that a 2-day inescapable foot shock results in aberrant dopamine neuron activity and related behavioral deficits in sensorimotor gating.^10,11^ Importantly, pharmacological inhibition of either the vHipp^11,42^ or PVT^22,43^ can restore dopamine system function in rodent models that display elevated dopamine neuron population activity. However, the role of vHipp-NAc and PVT-NAc projections in modulating dopamine neuron population activity following foot shock stress has yet to be determined. Both the vHipp and PVT project to multiple brain regions known to regulate dopamine neuron population activity, including the NAc and the medial prefrontal cortex (mPFC), warranting the specific investigation of these discrete pathways after stress.^44-46^

In the current experiments, we first examined how foot shock stress altered the firing rate and bursting patterns of neurons in the vHipp and PVT. Given that the PVT is known to display heightened activity in response to numerous stressors, we then examined stress-induced alterations in coordinated activity between the PVT and NAc. We provide evidence that coordinated activity between the PVT and NAc, but not between the PVT and mPFC, is increased after foot shock and that this may be a potential mechanism leading to aberrant VTA dopamine neuron population activity. Finally, we report that chemogenetic inhibition of either vHipp-NAc or PVT-NAc projections, following foot shock stress, restores dopamine system function. Taken together, we demonstrate that foot shock stress disrupts signaling between the PVT and NAc, a pathway known to increase dopamine neuron activity and that inhibiting either the vHipp-NAc or PVT-NAc pathways may be viable strategies to reverse stress-induced alterations in dopamine system function.

## METHODS AND MATERIALS

All experiments were performed in accordance with the guidelines outlined in the USPH Guide for the Care and Use of Laboratory Animals and were approved by the Institutional Animal Care and the Use Committees of UT Health San Antonio and the U.S. Department of Veterans Affairs.

### Two-Day Inescapable Foot Shock Stress

Male Sprague Dawley rats (250–300 g) were used for all experiments. Rats randomly assigned to the stress group were placed in a 30.5 × 25.4 × 30.5 cm^3^ square, sound-attenuated conditioning chamber with metal walls and a stainless-steel grid shock floor (Coulbourn Instruments), where each day they received 60 × 15 seconds 0.8-mA foot shocks with an inter-rial interval (ITI) of 30 seconds with a 25% deviation (±7.5 seconds). Control rats were handled daily but not exposed to conditioning chambers. Electrophysiological experiments were conducted 24–48 hours following inescapable stress.

### In Vivo Extracellular Recordings

Rats were anesthetized using chloral hydrate (8% in saline; 400 mg/kg, i.p.) and placed in a stereotaxic frame. Supplemental anesthesia was administered to maintain suppression of limb compression withdrawal reflex. Core body temperature of 37 °C was sustained using a thermostatically controlled heating pad (PhysioSuite, Kent Scientific Coorporation). Extracellular glass microelectrodes (impedance ~6–10 MΩ) were lowered into the PVT (AP −2.0 to −2.6, ML ±0.2 to ±0.4 mm from bregma, and DV −5.0 to −5.8 mm from the brain surface), the vHipp (AP −4.8 to −5.2, ML ±4.8 to ±5.2 mm from bregma, and DV −5.0 to −8.5 mm from the brain surface), or the VTA (AP −5.3 to −5.7, ML ±0.6 to ±1.0 mm from bregma, and DV −6.5 to −9.0 mm from the brain surface) using a hydraulic micro-positioner (Model 640, Kopf Instruments). Spontaneous activity of PVT and vHipp neurons was recorded by making multiple 6–9 vertical passes, separated by 100 µm (PVT) or 200 µm (vHipp), using the following filter settings: low-frequency cutoff: 100 Hz; high-frequency cutoff: 30 kHz (Model 3000, A-M Systems). The percentage of action potentials occurring in bursts within the vHipp and PVT was defined as the incidence of spikes within <20 ms between them; termination of the burst is defined by >60 ms between spikes.^47^ Putative pyramidal neurons in the vHipp were identified as neurons with firing frequencies less than 2 Hz, in accordance with previously published criteria.^48^^–^^50^ To identify and record putative dopamine neurons in the VTA, the following filter settings were used: low-frequency cutoff: 30 Hz; high-frequency cutoff: 30 kHz. Spontaneously active dopamine neurons were recorded using previously established electrophysiological criteria^51,52^ by making multiple 6–9 vertical passes, separated by 200 µm, throughout the VTA. Three parameters of dopamine neuron activity were measured and analyzed: the number of dopamine neurons firing spontaneously (population activity),^53^ basal firing rate, and proportion of action potentials occurring in bursts (defined as the incidence of spikes within <80 ms between them); termination of the burst is defined as the occurrence of an interspike interval of >120 ms.^51,52^ Electrophysiological analysis of vHipp, PVT, and VTA dopamine neuron activity was performed using commercially available computer software (LabChart version 8; ADInstruments) and analyzed with Prism software (GraphPad Software).

### Conscious Local Field Potential Recordings

Survival surgeries were performed in a semi-sterile environment under Isoflurane anesthesia (Fluriso, 2%–5% USP with oxygen flow at 1 L/min). Polyimide-insulated stainless-steel electrodes (P1Technologies) were unilaterally implanted into the PVT (AP −2.0, ML +0.4, DV −5.3 mm from bregma), the NAc (AP +1.3, ML +1.4, DV −7.6 mm from bregma), and the mPFC (AP +3.0, ML +0.6, DV −4.5 mm from bregma). A subset of animals received electrode implants in the central amygdala but were not evaluated for this study. Non-insulated stainless-steel ground wires were wrapped around anchor screws and all electrodes and ground wires were fixed in place with dental cement. All animals received postoperative ketoprofen (5 mg/kg, s.c.). After animals were allowed to recover for 2 weeks, baseline LFPs were recorded for 10 minutes, 3 days in a row, in awake, freely moving rats. Implanted electrodes were connected to a spring 6-channel connector cable (P1Technologies). Open filter settings (high-frequency cutoff: 300 Hz; low-frequency cutoff: 0.3 Hz) were used to record LFPs (Model 3000, A-M Systems). Following the third baseline recording, rats randomly assigned to the stress group received 2 days of inescapable foot shock and control animals were handled. Twenty-four hours following the last day of foot shock, 10-minute LFPs were recorded in awake, freely moving animals, within their home cage. Oscillatory activity was recorded using LabChart software (ADInstruments).

### Coherence Analysis

Oscillations in the PVT, NAc, and mPFC were quantified with commercially available computer software (LabChart version 7.1) using the following frequency ranges divisions: Delta: 0.3–4 Hz, Theta: 4–8 Hz, Alpha: 8–13 Hz, Beta: 13–30 Hz, Low Gamma: 30–55 Hz, High Gamma: 65–99 Hz. Gamma oscillations were separated into 2 groups to avoid electrical noise at 60 Hz. Coherent activity was analyzed using NeuroExplorer (Nex Technologies). Coherence values were calculated using a Fast Fourier Transformation (FFT) with the following parameters: maximum frequency 100 Hz, 128 frequency values, normalized to raw power spectral density (PSD). FFTs were performed on ten random 10-second epochs of LFP activity, within each brain region, across all frequency divisions. Coherence values are represented as the average value of the 10 epochs for each animal.

### Chemogenetic Inhibition of NAc and mPFC Afferents

Rats were anesthetized using Fluriso (2%–5% Isoflurane, USP with oxygen flow at 1 L/min) and placed in a stereotaxic apparatus. Core body temperature was maintained at 37 °C. Guide cannula (22-gauge) aimed at the PVT (AP: −2.0 mm, ML: ± 0.4, DV: −5.2 mm from bregma) or vHipp (AP: −4.8, ML: ±4.8, DV: −7.2 mm from bregma) were used to bilaterally inject 0.5μL (PVT) or 0.75μL (vHipp) of the Cre-dependent Gi inhibitory DREADD virus (pAAV-hSyn-DIO-hM4D(Gi)-mCherry; Addgene #44362-AAV2). Concurrently, bilateral cannula aimed at either the mPFC (AP +3.0, ML ±0.6, DV −4.5 mm from bregma) or NAc (AP +1.4, ML ±1.3, DV −7.6 mm from bregma) were used to inject 0.5 μL of the AAV2 retrograde-Cre (pENN-AAV-hSyn-HI-eGFP-Cre.WPRE.SV40; Addgene #105540-AAVrg) (Figures 3A and 4A). All animals received postoperative ketoprofen (5 mg/kg, s.c.). Rats were allowed 6–8 weeks to recover and to ensure maximal viral expression. To inactivate discrete neuronal projections, the DREADD agonist Compound 21 (3 mg/kg, i.p.; HelloBio #HB6124) was administered 20 minutes prior to recordings or water (i.p) as a control. The vehicle group was a mixture of animals that received an inhibitory DREADD, in either the PVT or vHipp, but a vehicle injection (water, i.p.) prior to in vivo recordings. The number of spontaneously active cells per track was not statistically different between the vehicle-treated animals. Representative images of Gi DREADD expression are shown in the PVT (Figure 3B) and the vHipp (Figure 4B).

### Statistical Analysis

All data were analyzed using SigmaPlot (Systat Software Inc.). vHipp and PVT firing rate data and PVT-NAc and PVT-mPFC coherence data were analyzed by *t*-test and VTA dopamine neuron activity data were analyzed by 2-way ANOVA (factors: stress × drug). Holm-Sidak was used for all post-hoc analyses, with significance determined at *P* < .05. All data are represented as the mean ± SEM, with *n* values representing the number of rats per group unless otherwise specified. Sample sizes were determined based on our previous studies.^49,54^

### Histology

A subset of rats was transcardially perfused immediately following electrophysiological recordings with saline (150 mL) followed by formaldehyde (150 mL; 4% w/v in phosphate-buffered saline [PBS]), then rapidly decapitated. Brains were extracted and post-fixed for at least 24 hours (4% formaldehyde in saline) and cryoprotected (10% w/v sucrose in PBS) until saturated. Histological verification of single-cell and LFP electrode tracks within the VTA, PVT, NAc, mPFC, and vHipp were performed in 25-μm coronal sections that were collected on a cryostat (Leica) and mounted onto gelatin-chrome alum-coated slides and stained with neutral red (0.1%) and thionin acetate (0.01%).^55^ Confirmation of DREADD and retrograde-Cre expression in either the vHipp or PVT were performed in 50-μm coronal sections in a subset of experimental and nonexperimental rats.

## RESULTS

### Foot Shock Stress Significantly Alters Bursting Activity, But Not Firing Rate, of vHipp and PVT Neurons

**Figure 1.**
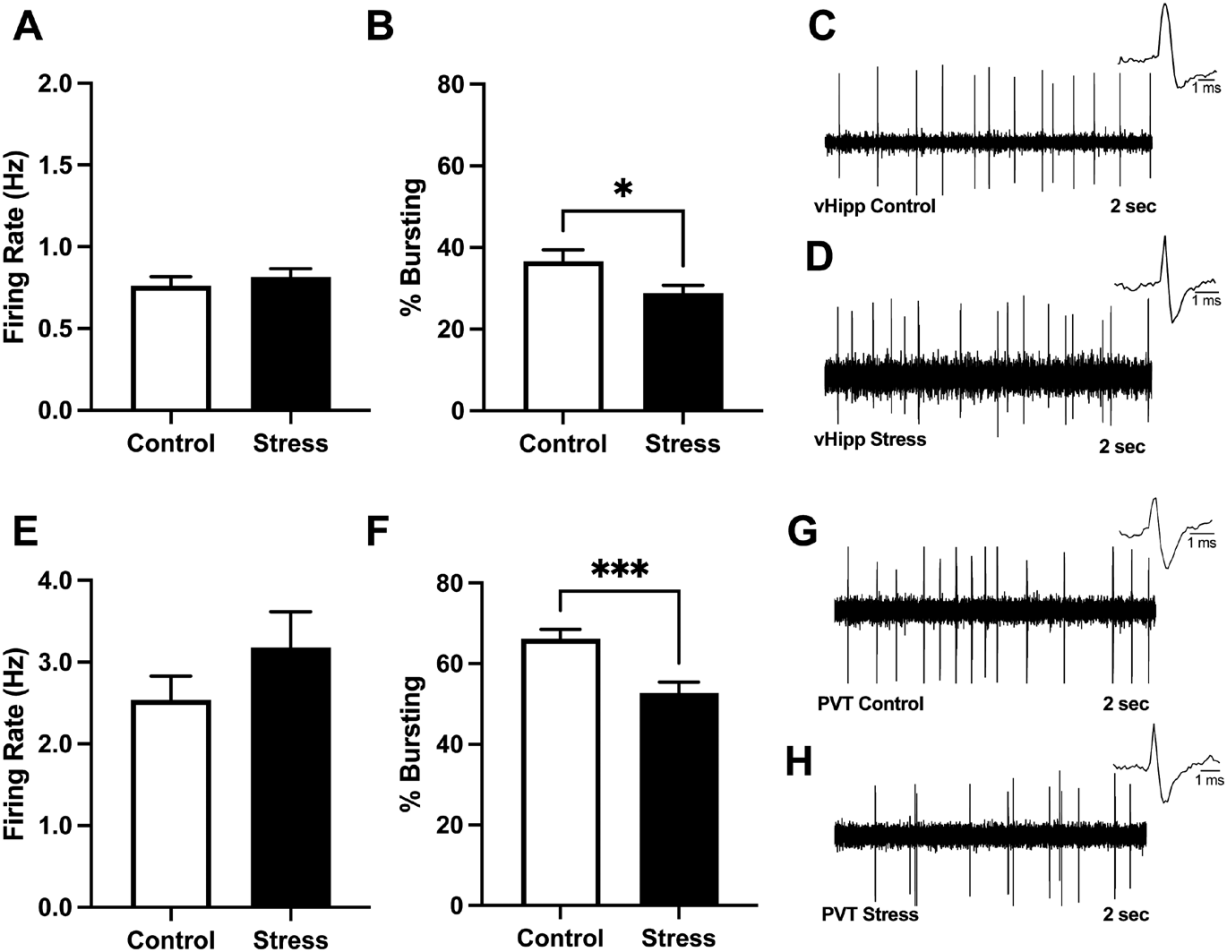
Inescapable foot shock has no effect on the firing rate of vHipp pyramidal neurons (A) but does significantly decrease the percentage of action potentials occurring in bursts (B). Representative traces from vHipp control (C), vHipp stress (D) are shown. The firing rate of PVT neurons was not significantly different following inescapable foot shock stress (E). The percentage of action potentials occurring in bursts is significantly reduced in PVT neurons after foot shock stress. Representative traces from PVT control (G) and PVT stress animals (H) are shown. * *P* < .05 compared to control. *** *P* < .001 compared to control.

Both pharmacological and chemogenetic activations of the vHipp or PVT have been shown to result in increased VTA dopamine neuron activity.^22^ Considering that the vHipp and PVT are stress sensitive, we posited that foot shock stress may increase VTA population activity by increasing the activity in these regions. Therefore, we utilized in vivo extracellular electrophysiology to examine how foot shock stress altered the bursting pattern and firing rate of neurons in the vHipp and PVT. Although an increase in vHipp firing rate has been observed in rodent models used to study psychosis,^49,56^ we found no differences in the firing rate of putative pyramidal cells within the vHipp following foot shock stress (Figure 1A; *t*-test; *t* = 0.696; *P* = .487; control = 105 neurons; 0.76 ± 0.6 Hz; stress = 115 neurons; 0.81 ± 0.5 Hz). However, we observed a significant decrease in the percentage of action potentials occurring in bursts (Figure 1B; *t*-test; *t* = 2.328; *P* = .021) within the vHipp of stressed animals (*n* = 108; 28.81% ± 1.97%) compared to controls (*n* = 92; 36.63% ± 2.81%). Previous research has shown that PVT is a stress-sensitive region that displays increased cFos expression and calcium transients in response to aversive stimuli.^31-37^ Although foot shock stress did not significantly increase the firing rate of PVT neurons compared to controls (Figure 1E; *t*-test; *t* = 1.218; *P* = .221; control = 110 cells; 2.54 ± 0.29 Hz; stress = 115 cells; 3.18 ± 0.43 Hz), foot shock stress did significantly decrease the percentage of action potentials occurring in bursts in the PVT (Figure 1F; *t*-test; *t* = 3.773; *P* = .002; control = 101 cells; 66.17%% ± 2.30%; stress = 113 cells; 52.77% ± 2.65%). Representative traces from control and stress animals are shown in Figure 1C, D, G, H.

### Coherent Neuronal Activity Increases in PVT-NAc, Not PVT-mPFC Projections, Following Foot Shock Stress

**Figure 2.**
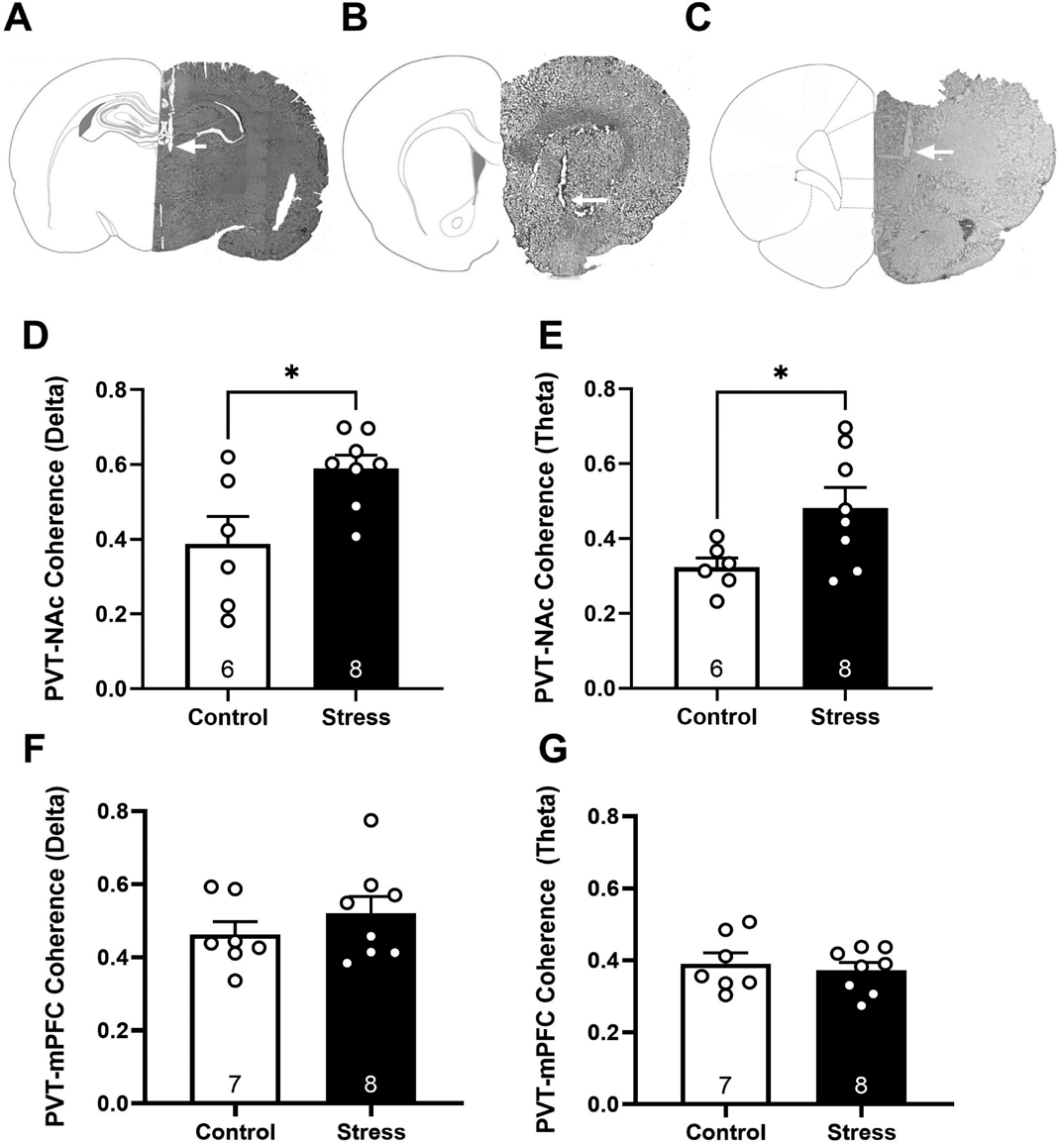
Foot shock stress increases coordinated activity between the PVT and NAc but not between the PVT and mPFC. Histological verification of electrode placement for the PVT (A), NAc (B), and mPFC (C) are shown. Arrows denote the electrode track. Foot shock stress significantly increased delta synchrony (D) and theta coherence (E) between the PVT and NAc. * *P* < .05 compared to control. No significant changes were observed in delta synchrony (F) or theta synchrony (G) between the PVT and mPFC.

Local field potential recordings can capture an integrative, holistic view of neuronal signaling within a brain region.^57,58^ Further, LFPs can be recorded simultaneously in multiple brain regions and then analyzed to determine how synchronous neuronal activity is coordinated between regions of interest. Increased coherent oscillatory activity between regions is thought to represent the enhanced transmission of information that is relevant for guiding behavior and perception.^59^ Exposure to foot shock stress has been shown to increase activity in PVT to NAc projections, quantified by cFos expression^34^; however, it is unknown how environmental stressors affect coordinated activity between these 2 regions. We simultaneously measured spontaneous LFP activity in the PVT, NAc, and mPFC to understand how foot shock stress influenced coordinated neuronal activity. Foot shock stress significantly increased delta coherence between the PVT and NAc (Figure 2D; *t*-test; *t* = 2.712; *P* = .02; control: 0.389 ± 0.07; stress: 0.589 ± 0.04; *n* = 6–8 rats per group). We also observed an increase in theta synchrony between the PVT and NAc, following foot shock stress (Figure 2E; *t*-test; *t* = 2.384; *P* = .03; control: 0.323 ± 0.02; stress: 0.482 ± 0.05; *n* = 6–8 rats/group). Unlike PVT-NAc projections, we found no differences in delta synchrony (Figure 2F; *t*-test; *t* = 0.9698; *P* = .35; control: 0.4623 ± 0.14; stress: 0.5201 ± 0.18; *n* = 7–8 rats per group) or theta synchrony (Figure 2G; *t*-test; *t* = 0.5220; *P* = .61; control: 0.3913 ± 0.13; stress: 0.3723 ± 0.10; *n* = 7–8 rats per group) between the PVT and mPFC. No differences were observed in alpha, beta, low gamma, or high gamma frequencies in either PVT-NAc or PVT-mPFC projections. Representative electrode tracks in the PVT, NAc, and mPFC are shown in Figure 2A–C.

### Chemogenetic Inhibition of the PVT-NAc, But Not PVT-mPFC, Pathway Restores Dopamine System Function Following Foot Shock Stress

**Figure 3.**
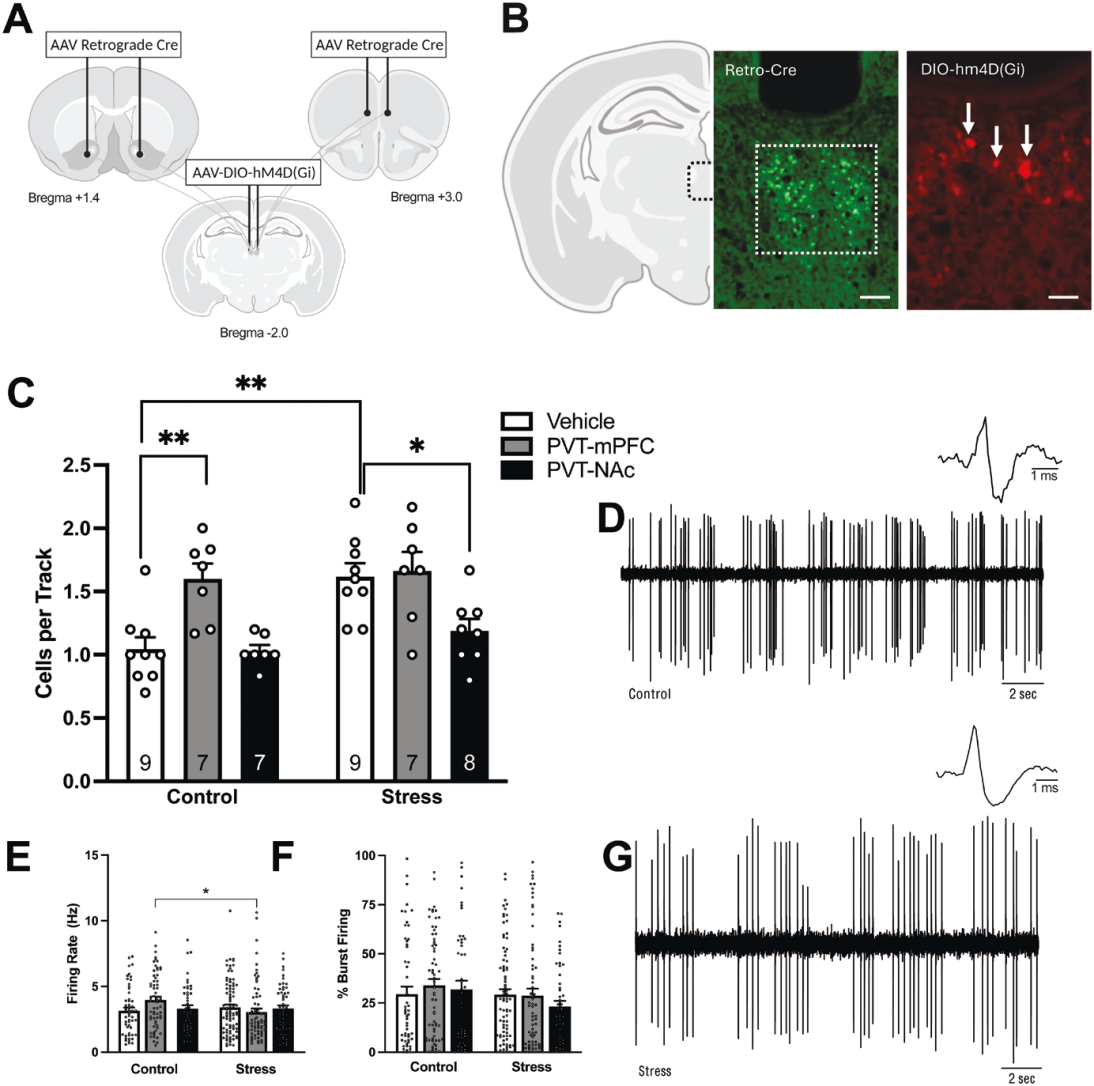
Inhibition of PVT–NAc projections restores dopamine system function following foot shock stress. (A) Schematic representation of concurrent injections of an AAV retrograde Cre virus into either the NAc or mPFC and Cre-dependent inhibitory Gi DREADD into the PVT. (B) Verification of retrograde Cre (left; scale bar represents 200 µm) and Gi DREADD (right; scale bar represents 50 µm) expression in the PVT. (C) The number of spontaneously active dopamine neurons per electrode track (population activity) was increased following foot shock stress. Chemogenetic inhibition of PVT-NAc projections restored dopamine system function. In control animals, inhibition of PVT-mPFC projections significantly increased population activity. Representative traces from control (D) and stress animals (G). (E) Significant differences in firing rate were observed between control/PVT–mPFC animals and stress/PVT–mPFC animals. (F) There were no significant differences in bursting pattern. *P < .05 compared to stress/vehicle. **P < .01 compared to control/vehicle.

Increased mesolimbic dopamine transmission has been correlated with psychosis symptom severity.^19,60^ Therefore, we assessed dopamine neuron activity using in vivo extracellular electrophysiology.^51,52^ Here, we determined if select inhibition of PVT-NAc projections restored dopamine system function following stress. Consistent with our previous reports, we found a main effect of foot shock stress (Figure 3C; 2-way ANOVA; factors: stress × target region inactivation; *F*_(1,46)_ = 9.147; *P* = .004; *n* = 7–9 rats/group). Foot shock stress animals displayed an increase in VTA dopamine neuron population activity compared to controls (Holm-Sidak: *t* = 4.078, *P* < .001; control/vehicle: *n* = 9; 1.04 ± 0.09 cells/track; stress/vehicle; 1.62 ± 0.11 cells/track). Importantly, inhibition of PVT-NAc projections, in foot-shock-stressed animals, significantly reversed aberrant dopamine neuron population activity compared to stress/vehicle animals (Holm-Sidak: *t* = 2.969; *P* = .010; 1.19 ± 0.09 cells/track) but inhibition of PVT-mPFC projections did not (Holm-Sidak: *t* = 0.2989; *P* = .767; 1.66 ± 0.15 cells/track). Representative traces are shown in Figure 3D, G. An unexpected increase in population activity was observed in control animals following chemogenetic inhibition of PVT-mPFC projections (Holm-Sidak: *t* = 3.701; *P* = .002; 1.60 ± 0.12 cells/track) compared to control/vehicle animals. In addition to population activity, we examined 2 other parameters of dopamine cell activity: firing rate and percentage of spikes fired in bursts. We observed significant differences in firing rate (Figure 3E; Holm-Sidak: *t* = 2.634, *P* = .009) when comparing control/PVT-mPFC (*n* = 66 neurons; 3.97 ± 0.26 Hz) and stress/PVT-mPFC animals (*n* = 70 neurons; 3.06 ± 0.26 Hz). No other significant differences were observed in the firing rate (control/veh: *n* = 54 neurons; 3.15 ± 0.25 Hz; stress/veh: *n* = 86 neurons; 3.40 ± 0.22 Hz; con/PVT-NAc: *n* = 46 neurons; 3.29 ± 0.28 Hz; stress/PVT-NAc: *n* = 52 neurons; 3.31 ± 0.25 Hz) or percentage of action potentials occurring in bursts in any of the groups (Figure 3F; control/veh: *n* = 54 neurons; 29.47% ± 3.82%; stress/veh: *n* = 86 neurons; 29.26% ± 2.69%; con/PVT-mPFC: *n* = 66 neurons; 33.96% ± 3.24%; stress/PVT-mPFC: *n* = 70; 28.81 ± 3.54; con/PVT-NAc: *n* = 46 neurons; 31.89% ± 4.46%; stress/PVT-NAc: *n* = 52 neurons; 23.14% ± 2.92%).

### Chemogenetic Inhibition of the vHipp-NAc, But Not vHipp-mPFC, Pathway Restores Dopamine System Function Following Foot Shock Stress

**Figure 4.**
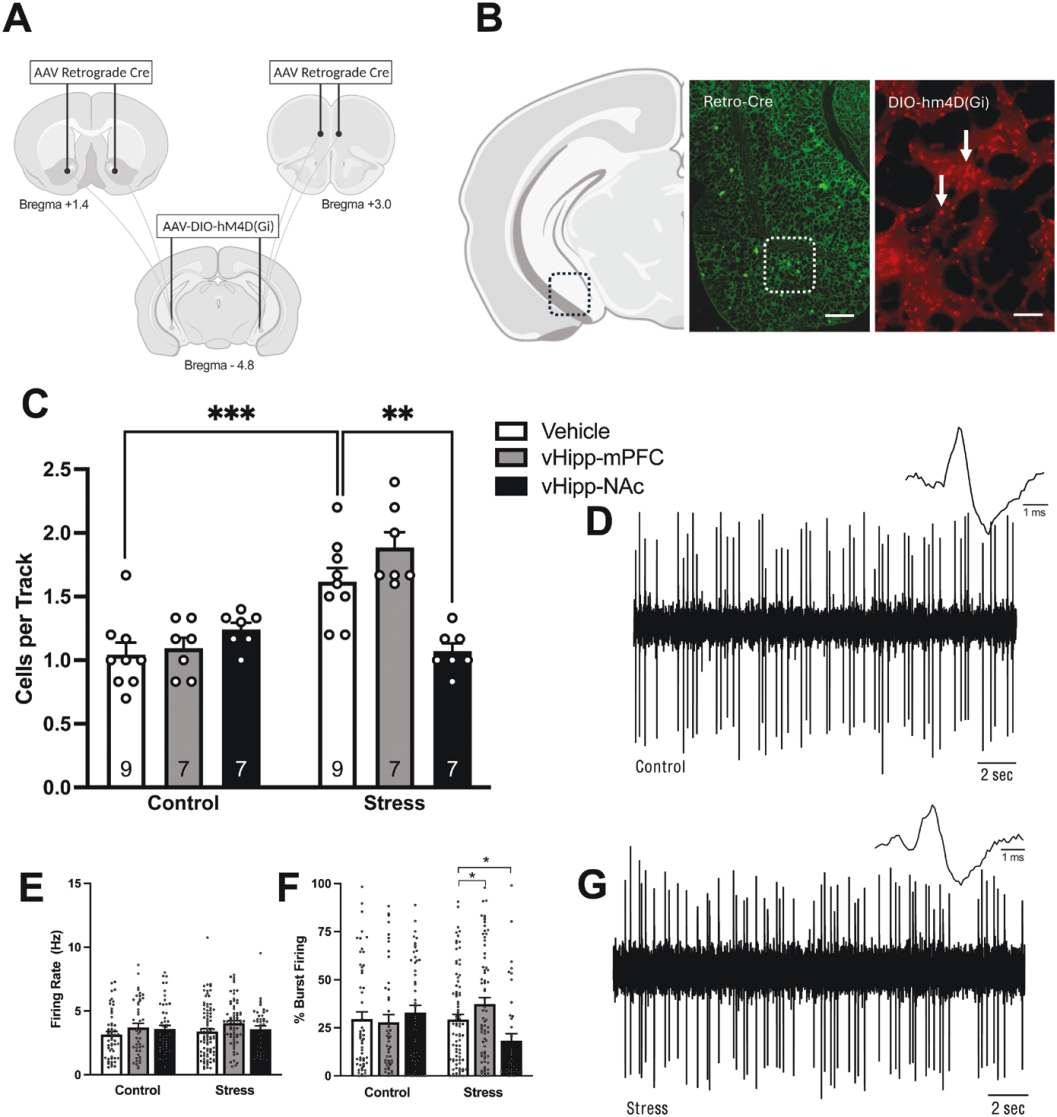
Inhibition of vHipp–NAc projections restores dopamine system function following foot shock stress. (A) Schematic representation of concurrent injections of an AAV retrograde Cre virus into either the NAc or mPFC and Cre-dependent inhibitory Gi DREADD into the vHipp. (B) Representative image of retrograde Cre (left; scale bar represents 500 µm) and Gi DREADD (right; scale bar represents 100 µm) expression in the vHipp. (C) The number of spontaneously active dopamine neurons per electrode track (population activity) was increased following foot shock stress. Chemogenetic inhibition of vHipp-NAc projections restored dopamine system function. Representative traces from control (D) and stress animals (G). (E) There were no significant differences in firing rate. (F) Bursting pattern was significantly increased in vHipp–mPFC animals compared to stress/vehicle while the bursting activity of vHipp-NAc animals was significantly decreased from stress/vehicle. *P < .05 compared to stress/vehicle. **P < .01 compared to stress/vehicle. ***P < .001 compared to control/vehicle.

Previous reports have shown that the PVT and vHipp provide convergent inputs to the NAc and work in concert to regulate VTA dopamine neuron activity.^22^ As detailed above, inhibition of PVT-NAc projections reversed foot shock stress increases in dopamine neuron population activity. Here, we determined if inhibition of vHipp-NAc projections could similarly restore dopamine system function following stress. Representative images of DREADD expression in the vHipp are shown in Figure 4B. We found a main effect of foot shock stress (Figure 4C; 2-way ANOVA; factors: stress × target region inactivation; *F*_(1,45)_ = 27.431; *P* < .001; *n* = 7–9 rats/group). Compared to controls, foot shock stress increased VTA dopamine neuron population activity (Holm-Sidak: *t* = 5.237, *P* < .001; control/vehicle: *n* = 9; 1.04 ± 0.09 cells/track; stress/vehicle; 1.62 ± 0.11 cells/track). Representative traces are shown in Figure 4D, G. In support of our hypothesis, inhibition of vHipp-NAc projections, following foot shock stress, significantly reversed dopamine neuron population activity compared to stress/vehicle animals (Holm-Sidak: *t* = 4.239; *P* < .001; 1.07 ± 0.06 cells/track). Conversely, inhibition of vHipp-mPFC projections in stressed animals had no effect on population activity, which remained significantly increased compared to vHipp-mPFC controls (Holm-Sidak: *t* = 5.791; *P* < .001; stress/vH-mPFC: 1.89 ± 0.10 cells/track; con/vH-mPFC: 1.10 ± 0.10 cells/track). We examined 2 other parameters of dopamine cell activity: firing rate and percentage bursting. No significant differences were observed in the firing rate (Figure 4E; control/veh: *n* = 54 neurons; 3.15 ± 0.25 Hz; stress/veh: *n* = 86 neurons; 3.40 ± 0.22 Hz; con/vHipp-mPFC: *n* = 46 neurons; 3.71 ± 0.32 Hz; stress/vHipp-mPFC: *n* = 71 neurons; 4.05 ± 0.21 Hz; con/vHipp-NAc: *n* = 51 neurons; 3.60 ± 0.26 Hz; stress/vHipp-NAc: *n* = 40 neurons; 3.52 ± 0.29 Hz). We did observe significant differences in the percentage of action potentials occurring in bursts within the stress groups. Inhibition of vHipp-NAc projections decreased burst firing compared to control animals (Figure 4F; 2-way ANOVA; factors: stress × target region inactivation; Holm-Sidak: *t* = 2.140; *P* = .05; stress/veh: *n* = 86 neurons; 29.26% ± 2.66%; stress/vHipp-NAc: *n* = 40 neurons; 18.21% ± 3.80%). Inhibition of vHipp-mPFC projections significantly increased burst firing compared to controls (Holm-Sidak; *t* = 2.248; *P* = .05; stress/vHipp-mPFC: *n* = 71; 39.16% ± 3.68%). No other significant differences in burst firing were observed (control/veh: *n* = 54 neurons; 29.47% ± 3.82%; con/vHipp-mPFC: *n* = 46 neurons; 27.89% ± 3.98%; con/vHipp-NAc: *n* = 51 neurons; 32.83% ± 3.88%).

## DISCUSSION

Stress is a common risk factor for many psychiatric illnesses^1^ and it has been observed that disorders related to periods of profound or prolonged stress, such as PTSD, have a high co-incidence of psychosis symptoms, that is, hallucinations and delusions.^13-17^ Symptoms of psychosis are attributed to an increase in mesolimbic dopamine transmission and various environmental stressors have been shown to increase mesolimbic dopamine activity minutes to days after exposure to the aversive stimuli.^4-6,10-12,19,22^ Specifically, exposure to 2-day inescapable foot shock results in elevated VTA dopamine neuron population activity, which is thought to underlie symptoms of psychosis.^10,11^ Although aberrant dopamine neuron activity is observed, recent evidence suggests that stress-induced pathology may lie in brain regions that provide afferent regulation of the dopamine system, such as the vHipp or PVT.^10,22^

The vHipp is responsive to physical and psychological stress and is known to be altered in both psychosis and stress-based illnesses independently. Specifically, hippocampal abnormalities, such as reduced volume and functionality are common in both PTSD and psychosis patients.^23,24,28,29^ Evidence suggests that this reduced functionality is associated with exaggerated hippocampal activation,^24,25^ although it should be noted that hippocampal activity may increase or decrease following stress, depending on various factors.^30^ Previous research has demonstrated in rodent models used to study psychosis, the firing rate of vHipp neurons is elevated^49,56,61^ and this enhanced vHipp activity can drive aberrant dopamine neuron activity in the VTA and produce psychosis-like alterations in behavior.^21,22,62^ Thus, we hypothesized that, in our stress-based model, enhanced VTA activity may be driven by increased vHipp neuron firing rate. However, we observed no differences in the firing rate of vHipp pyramidal neurons, suggesting that the pathology contributing to increased VTA dopamine neuron population activity following foot shock stress may lie in the PVT, another afferent regulator of the dopamine system.

The PVT also displays heightened activity, as measured by cFos or calcium imaging, following exposure to aversive stimuli, such as foot shock or restraint stress.^31-36^ However, there are few data directly examining alterations in the firing rates of PVT neurons following foot shock stress. Using in vivo extracellular electrophysiology, we found no significant increases in PVT neuron activity following stress when compared to control animals. While these data appear to contradict previous work showing increased cFos expression in the PVT and specifically in PVT cells projecting to the NAc and mPFC, following foot shock, it is possible that our sampling of cells across the entire anterior-posterior axis of the PVT, within the same animals masked potentially significant differences. The PVT can be divided into distinct anatomical subregions with unique expression profiles and projection targets.^44,63^ Previous research using calcium imaging to examine single-cell activity in the PVT has garnered mixed results, showing both excitatory and inhibitory responses to similar environmental stimuli.^64-66^ Neurons in the PVT can be divided into 2 distinct types, type I and type II, based on genetic markers, projection targets, and functional relevance.^63^ Specifically, type I neurons show increased activity in response to aversive stimuli and decreased activity to rewarding stimuli, while type II neurons decrease activity in response to both aversive and rewarding stimuli, suggesting type II neurons encode salience, regardless of valence.^63^ Thus, it is possible that the increase in VTA dopamine neuron activity is driven by type I neurons in the PVT following foot shock stress. While the methods used here were unable to distinguish between these cell types, future research should aim to examine firing rate alterations in discrete PVT cell types following stress.

Previous research has demonstrated various stressors can significantly influence multiple parameters of neuronal firing in the hippocampus and PVT, beyond that of firing rate. Indeed, chronic stress has been shown to alter burst length and increase intra-burst intervals in hippocampal neurons.^67^ Further, oxidative stress has been shown to decrease the burst length of thalamic neurons.^68^ To further understand changes in activity of vHipp and PVT neurons following foot shock stress, we examined the bursting pattern of these neurons, and indeed, observed a disruption in the firing pattern of these cells. Here, we show the effects of acute stress result in a decrease in both vHipp and PVT bursting activity, similar to previous research. Together, our data demonstrate a disruption in neuronal signaling within the vHipp and PVT, following foot shock stress.

Although overt increases in firing rate were not observed in the PVT, we hypothesized a subset of PVT neurons display heightened activity following foot shock stress. Given that the single-cell recordings were conducted in anesthetized animals, we sought to examine coordinated activity, between the PVT and downstream targets, in awake, freely moving rats, before and after stress. Previous work has demonstrated that increased glutamatergic transmission specifically in projections from the PVT to the NAc can increase dopamine neuron population activity.^22^ Therefore, we hypothesized that PVT neurons projecting to the NAc may be susceptible to stress-induced increases in activity. To examine if stress-induced changes were unique to PVT-NAc projections or reflective of global changes in PVT synchrony with other target regions, we also quantified coherent activity between the PVT and mPFC. We examined if foot shock stress increased coordinated activity between the PVT and these two regions, in awake, freely moving animals. Indeed, we observed an increase in delta and theta synchrony between the PVT and NAc in foot shock stress animals but saw no changes in delta or theta synchrony between the PVT and mPFC. The NAc is a critical region for action selection and previous research has demonstrated that delta coupling between the NAc and mPFC occurs during decision-making in both human and animal models^69^; however, this is the first report, of our knowledge, to demonstrate an increase in PVT-NAc delta coherence following a stressful event. Future studies will elucidate the role of PVT-NAc delta synchrony in guiding motivated behaviors. Additionally, we observed an increase in theta coherence between the PVT and NAc. Theta frequencies are often associated with increased vigilance and attention,^70,71^ suggesting that foot shock stress may contribute to heightened arousal by increasing theta oscillatory activity between the PVT and NAc.

Although the underlying neurobiological mechanisms contributing to psychiatric diseases are complex, a consistent finding is that the PVT displays aberrant activation following stressful and aversive events.^31-36,72^ The PVT is extensively connected to brain regions that regulate mood, emotion, and motivated behaviors, including the NAc and the mPFC. Importantly, these distinct PVT projections vary in their functional relevance, as PVT-NAc projections are involved in arousal^73^ and reward-seeking behaviors^65^ while PVT–mPFC projections appear critical for the formation and extinction of conditioned fear.^72^ Although these projections appear to be unique in function, they both demonstrate increased activation following foot shock stress,^34^ strengthening the notion of the PVT as an integrative hub that responds to environmental stressors and influences behavior through thalamostriatal and thalamocortical connections. Of importance to the current study is the ability of projections from the PVT to NAc or mPFC to modulate dopamine neuron activity. We have previously demonstrated that chemogenetic activation of glutamatergic projections from the PVT to the NAc, but not PVT-mPFC, can significantly increase dopamine neuron population activity.^22^ Further, in a rodent model used to study dopamine system dysfunction, pharmacological inhibition of the PVT can restore dopamine system function.^22,43^ These data suggest hyperactivity occurs in PVT-NAc projections after foot shock and that selective inhibition of PVT-NAc projections may restore dopamine system function. In the current experiments, we utilized a chemogenetic approach to selectively inhibit PVT-NAc or PVT-mPFC projections following foot shock stress and measured VTA dopamine neuron population activity. Given that only a small portion (10-20%) of PVT neurons collateralize to innervate both the NAc and the mPFC, it is likely that the chemogenetic approach utilized resulted in selective inhibition of these distinct projections.^39^ It is important to note that expression of the Gi DREADD within PVT and vHipp cells that targeted either the mFPC or NAc was confirmed in a subset of experimental rats but to increase the rigor of our findings, we performed identical viral surgeries in a cohort of non-experimental rats. Given that some of our regions of interest are relatively small structures, we verified viral expression of both the retrograde Cre and Gi DREADD were within these desired regions.

Consistent with previous reports,^10,11^ we found that foot shock stress significantly increases VTA dopamine neuron population activity. As expected, inhibition of PVT-NAc projections was able to reverse foot shock-induced increases in population activity, while inhibition of PVT-mPFC projections had no effect on population activity in stressed animals. These data provide further support that increased activation of PVT-NAc following foot shock stress contributes to elevated dopamine neuron population activity. Interestingly, we found that inhibition of PVT-mPFC increased dopamine neuron population activity in control animals. PVT projections to the mPFC form synaptic connections with inhibitory interneurons and help facilitate feedforward inhibition.^74,75^ Our laboratory has previously shown that disinhibition of the mPFC, using the GABA_A_ receptor antagonist, bicuculline, can significantly increase dopamine neuron population activity.^76^ Therefore, inhibition of PVT-mPFC projections in healthy animals likely resulted in increased dopamine neuron population activity by disinhibiting mPFC activity, although this remains to be established.

The vHipp and PVT have been shown to work in concert to regulate dopamine neuron activity, through convergent inputs to medium spiny neurons in the NAc. These convergent inputs are thought to contribute to the bistable membrane potential of MSNs within the NAc, which display a relatively depolarized membrane potential (approximately −60 mV) in the “UP” state and a relatively hyperpolarized membrane potential (approximately −75 mV) in the “DOWN” state. Excitatory inputs to the NAc from the vHipp contribute to UP states, and PVT inputs are thought to drive action potential generation.^22^ Thus, these 2 regions together are necessary to regulate VTA dopamine neuron activity, even if the foot-shock-induced pathology lies in only one of these regions. Therefore, we additionally examined if chemogenetic inhibition of vHipp-NAc projections could restore dopamine system function similarly to PVT-NAc inhibition. We found that, consistent with previous literature^77^ chemogenetic inhibition of vHipp projections to the NAc, and not the mPFC, was sufficient to reverse increases in VTA dopamine neuron population activity. Thus, although the two days of inescapable foot shock model does not appear to increase excitatory drive in the vHipp–NAc pathway, decreasing activity in this pathway can still normalize dopamine system function, presumably by decreasing the likelihood of MSNs existing in a UP state.

In the current experiments, we demonstrate that foot shock increases synchronized activity between the PVT and NAc, which likely contributes to elevated VTA dopamine neuron population activity. Interestingly, chemogenetic inhibition of either vHipp–NAc or PVT–NAc projections can restore dopamine system function after foot shock stress. These data suggest that inhibition of either vHipp or PVT activity can overcome neuropathological changes thought to underlie psychosis-like behavior. This study provides evidence in support of PVT–NAc projections as a potential circuit that contributes to symptoms of psychosis following stress but that targeting either the vHipp or PVT may be a viable treatment option for PTSD and comorbid psychosis.

## Data Availability

The data underlying this article will be shared on reasonable request to the corresponding author.

## Interest Statement

Dr. Lodge is co-inventor of a patent application covering novel analgesics and has an active collaboration with Sosei-Heptares, these are both unrelated to the work described in the current manuscript. The other authors declare no competing financial interests.
